# Omicron Wave SARS-CoV-2 Diagnosis: Evaluation of Saliva, Anterior Nasal, and Nasopharyngeal Swab Samples

**DOI:** 10.1128/spectrum.02521-22

**Published:** 2022-11-01

**Authors:** Marion Migueres, Jean-Michel Mansuy, Sandrine Vasseur, Nicolas Claverie, Catherine Lougarre, Françoise Soulier, Pauline Trémeaux, Jacques Izopet

**Affiliations:** a CHU Toulouse, Hôpital Purpan, Institut fédératif de Biologie, Laboratoire de virologie, Toulouse, France; b Institut Toulousain des Maladies Infectieuses et Inflammatoires (Infinity), INSERM UMR1291-CNRS UMR5051, Toulouse, France; c Université Toulouse III Paul-Sabatier, Toulouse, France; d CHU Toulouse, Hôpital Purpan, Centre de prélèvement COVID, Toulouse, France; City University of Hong Kong

**Keywords:** SARS-CoV-2, COVID-19, Omicron, saliva, anterior nasal swab, RT-PCR, diagnostic

## Abstract

The Omicron variant differs from earlier strains of SARS-CoV-2 in the way it enters host cells and grows *in vitro*. We therefore reevaluated its diagnosis using saliva, nasopharyngeal swab (NPs), and anterior nasal swab (ANs) specimens from 202 individuals (64.9% symptomatic) tested at the Toulouse University Hospital SARS-CoV-2 drive-through testing center. All tests were done with the Thermo Fisher TaqPath COVID-19 reverse transcription-PCR (RT-PCR) kit. Overall, 92 subjects (45.5%) had one or more positive specimens. Global sensitivities of saliva, NPs, and ANs were 94.6%, 90.2%, and 82.6%, respectively. Saliva provided significantly greater sensitivity among symptomatic patients tested within 5 days of symptom onset (100%) than did ANs (83.1%) or NPs (89.8%). We obtained follow-up samples for 7/20 individuals with discordant results. Among them, 5 symptomatic patients were diagnosed positive on saliva sample only, soon after symptom onset; NPs and ANs became positive only later. Thus, saliva samples are effective tools for the detection of the Omicron variant. In addition to its many advantages, such as improved patient acceptance and reduced cost, saliva sampling could help limit viral spread through earlier viral detection.

**IMPORTANCE** Diagnostic testing for SARS-CoV-2 is an essential component of the global strategy for the prevention and control of COVID-19. Since the beginning of the pandemic, numerous studies have evaluated the diagnostic sensitivity of different respiratory and oral specimens for SARS-CoV-2 detection. The pandemic has been since dominated by the emergence of new variants, the latest being the Omicron variant characterized by numerous mutations and changes in host tropism *in vitro* that might affect the diagnostic performance of tests depending on the sampling location. In this prospective study, we evaluated the clinical performance of NPs, ANs, and saliva for SARS-CoV-2 diagnosis during the Omicron wave. Our results highlight the effectiveness of saliva-based RT-PCR for the early detection of the Omicron variant. These findings may help to refine guidelines and support the use of a highly sensitive diagnostic method that allows earlier diagnosis, when transmission is the most critical.

## INTRODUCTION

Although nucleic acid amplification tests on nasopharyngeal swab (NPs) samples have long been considered the gold standard for detecting SARS-CoV-2, the Centers for Disease Control and Prevention (CDC) have also authorized the use of upper respiratory tract specimens such as nasal midturbinate swab (MTs), anterior nasal swab (ANs), and saliva specimens (1).

ANs and saliva have several advantages over NPs. NPs must be collected by trained health care personnel wearing suitable protective equipment, and patients find the process uncomfortable, which can reduce compliance. Collecting ANs and saliva samples is noninvasive and painless and can be easily done by patients themselves. Many studies that compared the sensitivities of ANs and saliva to those of NPs at the beginning of the pandemic obtained discordant results (2–5). However, review of the literature and meta-analyses now indicate that both saliva and ANs have similar or acceptable sensitivities compared to NPs (6–9).

The COVID-19 pandemic has been dominated by waves of new SARS-CoV-2 variants. First came the Alpha, Beta, Gamma, and Delta variants; these have now been replaced by the Omicron variant and its sublineages (10). This variant is more transmissible (11) and has a greater immune escape capacity (12, 13) and a less severe outcome (14) than earlier variants. While some have stressed the importance of reevaluating the suitability of different specimens for diagnosing new variants (15), the sensitivity of saliva and ANs samples for detecting the Omicron variant has not been thoroughly examined (16, 17). One recent study suggested that saliva samples were more suitable for detecting the Omicron variant than the Delta variant (17). *Ex vivo* studies have also demonstrated that the Omicron variant replicates more efficiently in bronchial tissue than in lungs. The preferential replication in the host upper respiratory tract cells (18) could affect diagnostic tests, depending on the sample origin.

This study investigates the sensitivities of saliva, ANs, and NPs samples for detecting the SARS-CoV-2 Omicron variant.

## RESULTS

We enrolled 202 individuals (average age, 35.2 years; range, 16 to 74 years; 45% male) who provided saliva, NPs, and ANs samples. At the time of sampling, 131 (64.9%) were symptomatic and 71 (35.1%) were asymptomatic. Most of them had been vaccinated: 69 (34.2%) with 2 doses and 116 (57.4%) with 3 doses; 17 (8.4%) were unvaccinated. Of the 202 subjects, 72 (35.6%) tested positive for all 3 specimens, while 92 (45.5%) had at least one positive sample (Table 1). The SARS-CoV-2 Omicron BA.1 strain was detected in 65/92 subjects, and the Omicron BA.2 strain was detected in 24/92. The viral loads of 3 individuals were too low for determining the SARS-CoV-2 variant sublineage.

**TABLE 1:** Subject characteristics

Characteristic	All samples negative	All samples positive	Discordant results
No.	110	72	20
Age, yr			
Median [IQR^*a*^]	30.5 [23.8–45.3]	31 [23.3–46]	34 [25–45.8]
Mean (SD)	34.9 (13.7)	35 (13.2)	37.9 (14.7)
Male, no. (%)	47 (42.7)	38 (52.8)	6 (30)
Symptomatic, no. (%)			
No	50 (45.5)	13 (18.1)	8 (40)
Yes	60 (54.5)	59 (81.9)	12 (60)
Symptom onset/diagnosis, no. (%)			
<5 days	49 (81.7)	49 (83.1)	10 (83.3)
≥5 days	11 (18.3)	10 (16.9)	2 (16.7)
SARS-CoV-2 variant, no. (%)			
Omicron BA.1		51 (70.8)	14 (82.4)
Omicron BA.2		21 (29.2)	3 (17.6)
Vaccination, no. (%)			
Unvaccinated	10 (9.1)	6 (8.3)	1 (5)
Primary (2 doses)	36 (32.7)	24 (33.3)	9 (45)
Primary + booster	64 (58.2)	42 (58.3)	10 (50)

a IQR, interquartile range.

We detected SARS-CoV-2 in the saliva samples of 87 subjects, in the NPs of 83, and in the ANs of 76 (Table 2). The overall sensitivity of saliva samples (94.6% [87.8 to 98.2]) was greater than that of ANs samples (82.6% [73.3 to 89.7]; *P* = 0.018) but not different from that of NPs samples (90.2% [82.2 to 95.4]; *P* = 0.41).

**TABLE 2:** Virus RNA in saliva, NPs, and ANs samples and sensitivities

Subject or virus	No. with positive specimen result:						Sensitivity (%)		
	Saliva^+^/AN^+^/NP^+^	Saliva^+^/AN^−^/NP^+^	Saliva^+^/AN^−^/NP^−^	Saliva^+^/AN^+^/NP^−^	Saliva^−^/AN^+^/NP^+^	Saliva^−^/AN^−^/NP^+^	Saliva	AN	NP
Global (*n* = 92)	72	6	8	1	3	2	94.6	82.6	90.2
Asymptomatic (*n* = 21)	13	2	2	1	1	2	85.7	71.4	85.7
Symptomatic <5 days (*n* = 59)	49	4	6	0	0	0	100	83.1	89.8
Symptomatic ≥5 days (*n* = 12)	10	0	0	0	2	0	83.3	100	100
BA.1 (*n* = 65)	51	4	6	1	2	1	95.4	83.1	89.2
BA.2 (*n* = 24)	21	1	1	0	1	0	95.8	91.7	95.8

The 92 SARS-CoV-2-positive individuals included 21 who were asymptomatic, 59 who were symptomatic for less than 5 days, and 12 who had symptoms for more than 5 days (Table 1). The samples from asymptomatic and symptomatic individuals diagnosed more than 5 days after symptom onset all had similar sensitivities (Table 2). The sensitivity of saliva samples (100% [93.9 to 100]) from symptomatic individuals tested during the first 5 days of symptoms was greater than that of ANs (83.1% [71.0 to 91.6]; *P* = 0.001) or NPs (89.8% [79.2 to 96.2]; *P* = 0.027) samples from these subjects. Moreover, saliva sensitivity was higher in individuals sampled during the first 5 days of symptoms (100% [93.9 to 100]) than afterward (83.3% [54.0 to 96.5]; *P* = 0.026). In contrast, no significant difference was observed according to sampling time after symptom onset for both NPs (*P* = 0.58) and ANs (*P* = 0.19). Saliva, ANs, and NPs were all similarly sensitive for detecting the Omicron sublineages BA.1 and BA.2 (Table 2).

**FIG 1:**
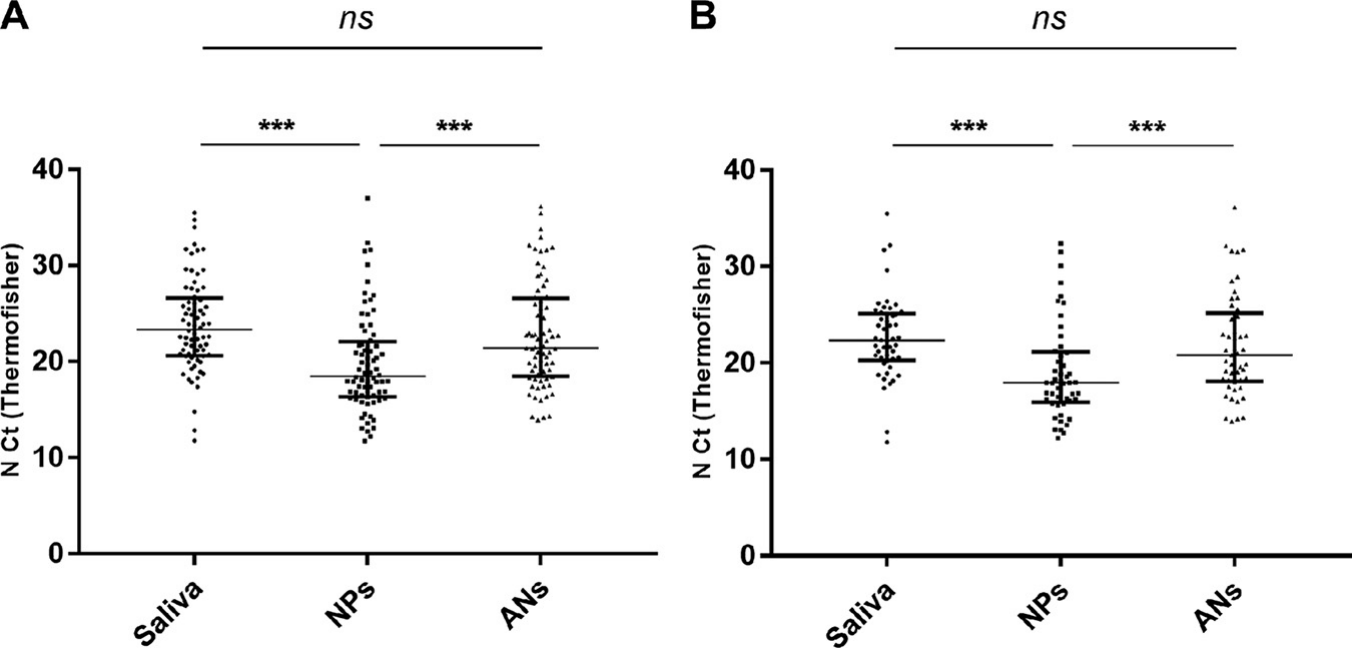
*C_T_* values for saliva, NPs, and ANs samples from 72 subjects positive for all 3 specimens (A) and from 49 symptomatic subjects diagnosed <5 days post-symptom onset for all 3 specimens (B). Data are medians plus interquartile ranges. ns, not significant. ***, *P* < 0.001.

Comparison of threshold cycle (*C_T_*) values between paired positive specimens demonstrated that NPs *C_T_* values were significantly lower than those of both saliva (median *C_T_* difference = 4.9; *P* < 0.001) and ANs (median *C_T_* difference = 2.2; *P* < 0.001), while those of saliva and ANs were not significantly different (*P* = 0.29) (Fig. 1A). The findings for symptomatic patients diagnosed within 5 days of symptom onset were similar (Fig. 1B).

**FIG 2:**
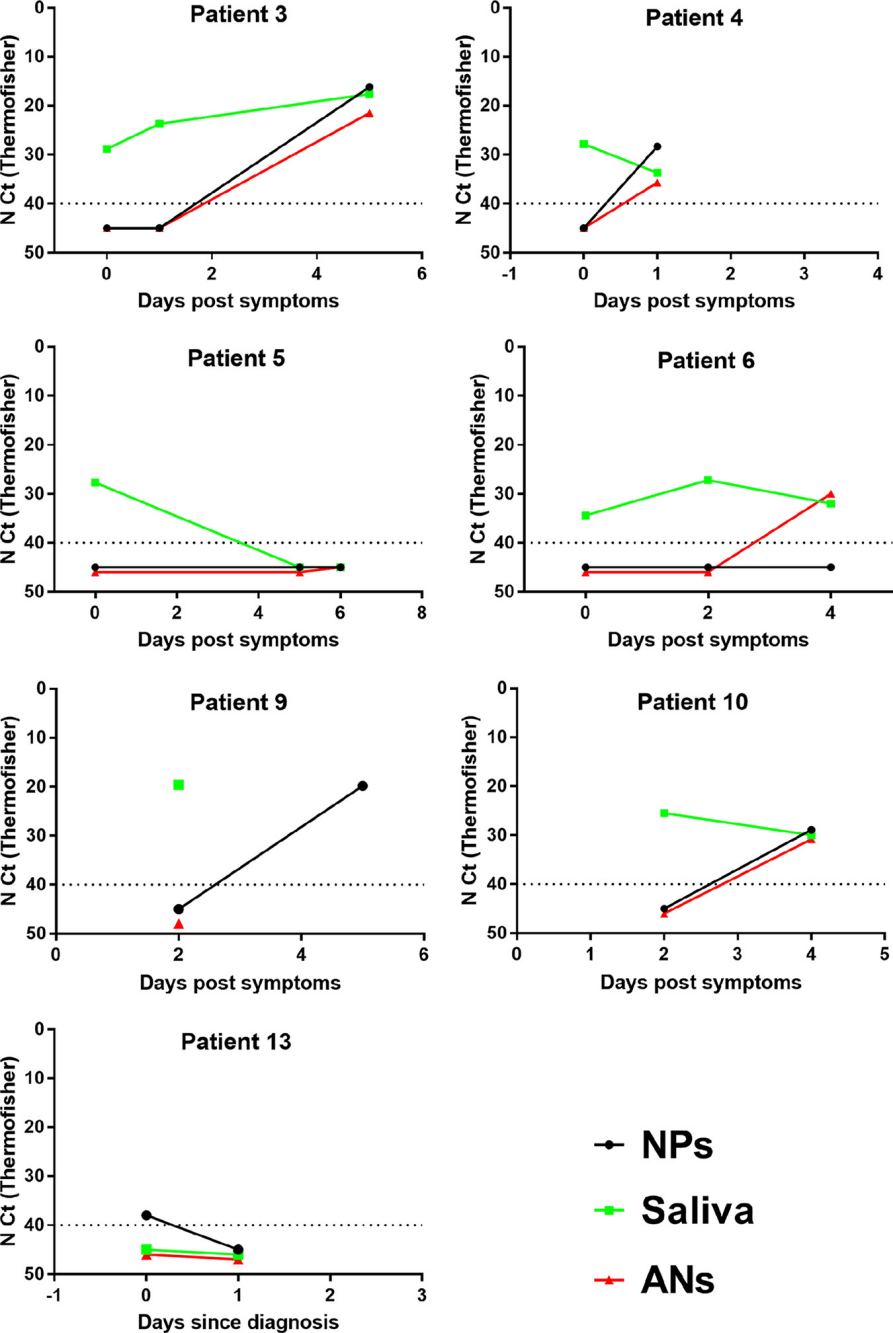
NPs, saliva, and ANs results for followed-up subjects.

Table 3 shows the characteristics of the 20 SARS-CoV-2-positive individuals who gave discordant results. They included 5 who were recovering from a previous SARS-CoV-2 infection. We followed up 7 of the 15 subjects with a primary diagnosis (Table 3 and Fig. 2). One asymptomatic subject (subject 13) tested positive for only the NPs sample on day 0 while testing negative for all 3 samples on day 1. The remaining 6 subjects were all symptomatic individuals whose saliva samples alone tested positive, early (<5 days) in their infection. The NPs and/or ANs samples of five of them were positive later, although all 3 samples from one (subject 5) tested negative the next day.

**TABLE 3:** Subjects with discordant results

Patient	Age (yr)	Sex^*a*^	Symptoms, onset/diagnosis	No. of vaccination doses	NPs (N *C_T_*)	ANs (N *C_T_*)	Saliva (N *C_T_*)	Previous infection	Follow-up
1	59	F	Symptomatic 0–1 day	3	+ (33.4)	−	+ (31.0)	No	No
2	23	M	Symptomatic 0–1 day	2	+ (32.0)	−	+ (20.3)	No	No
3	33	F	Symptomatic 0–1 day	2	−	−	+ (28.8)	No	Yes
4	35	F	Symptomatic 0–1 day	2	−	−	+ (27.7)	No	Yes
5	33	F	Symptomatic 0–1 day	2	−	−	+ (27.7)	No	Yes
6	35	F	Symptomatic 0–1 day	3	−	−	+ (34.3)	No	Yes
7	38	M	Symptomatic 0–1 day	3	+ (27.8)	−	+ (25.4)	No	No
8	45	F	Symptomatic 2–3 days	2	+ (25.1)	−	+ (13.6)	No	No
9	32	M	Symptomatic 2–3 days	2	−	−	+ (19.6)	No	Yes
10	23	M	Symptomatic 2–3 days	3	−	−	+ (25.5)	No	Yes
11	69	F	Symptomatic >8 days	3	+ (34.3)	+ (34.7)	−	10 days before	No
12	46	F	Symptomatic >8 days	0	+ (29.6)	+ (32.1)	−	9 days before	No
13	25	F	Asymptomatic	3	+ (38.0)	−	−	No	Yes
14	22	F	Asymptomatic	3	+ (16.9)	−	+ (21.4)	No	No
15	21	F	Asymptomatic	2	−	+ (36.7)	+ (36.8)	2 wk before	No
16	65	F	Asymptomatic	2	−	−	+ (32.8)	3 wk before	No
17	56	M	Asymptomatic	3	+ (22.6)	+ (25.7)	−	No	No
18	30	F	Asymptomatic	3	+ (34.2)	−	+ (27.9)	No	No
19	43	F	Asymptomatic	3	+ (30.3)	−	−	No	No
20	25	M	Asymptomatic	2	−	−	+ (31.9)	5 days before	No

a F, female; M, male.

## DISCUSSION

While many studies have evaluated the sensitivity of saliva and nasal swab specimens for detecting SARS-CoV-2 at the beginning of the pandemic (6–9), very few have reevaluated their performance since the emergence of the new SARS-CoV-2 variants (15), especially the Omicron variant (16, 17). Our prospective study provides further evidence of the high performance of SARS-CoV-2 diagnosis on saliva samples during the Omicron wave and highlights the higher capacity of saliva samples than of nasopharyngeal and nasal swab samples to detect Omicron variants at an early stage of infection.

Few published studies have compared nasal, nasopharyngeal, and saliva samples taken at the same time (19, 20). Most of them evaluate either the sensitivity of the saliva sample or the sensitivity of the nasal sample compared to the reference nasopharyngeal sample. The overall sensitivities of saliva (94.6%), ANs (82.6%), and NPs (90.2%) were similar. Our ANs performance data are also similar to those obtained before the emergence of the Omicron variant, with an acceptable sensitivity but slightly lower than nasopharyngeal sampling (6).

The results of published studies on the sensitivity of tests on saliva samples are conflicting, probably due to differences in the populations tested, the post-symptom-onset sampling time, or even how the saliva was collected and processed (4, 5, 7, 8, 21). Our data agree with those quoted in literature reviews and meta-analyses: the sensitivities of saliva and NPs samples are similar (7, 8). However, we report a higher performance of saliva samples than that in our previous study performed before the emergence of the variants of concerns (22). We previously reported a saliva sensitivity of 80%, lower than that for NP samples (96.4%), although we used similar saliva collection and pretreatment methods (22). The study populations were similar and included both asymptomatic and symptomatic subjects tested at the Toulouse University Hospital COVID center. The sensitivity of saliva samples varies greatly depending on how soon after infection onset they are taken, with early samples being more sensitive (23, 24). The October 2020 study included a greater proportion of symptomatic patients tested within 5 days of symptom onset (74.5%) than in the present study (64.1%), while fewer symptomatic subjects (9.1%) were tested over 5 days after symptom onset than in the present study (13%) (22). Although it is difficult to compare the results of our two distinct studies, these elements might suggest a better performance of saliva samples for detecting the RNA of Omicron variants than for that of previous strains and thus support recently published data (17).

Omicron variants have been assigned to different sublineages, such as BA.1 and BA.2, that differ in their transmissibility (25, 26). Why the BA.2 variant is more transmissible than BA.1 remains unclear. As there is evidence that established diagnostic methods should be reevaluated with the emergence of new variants (15), we compared the sensitivities of saliva, ANs, and NPs samples from Omicron BA.1- and BA.2-infected individuals but found no significant differences within this small group.

Our data confirm the influence of the time between sampling and infection on the sensitivity of salivary diagnosis; sensitivity was 100% for symptomatic patients sampled during the first 5 days but only 83.3% for samples taken long after symptom onset. We also find that saliva samples are more sensitive than NPs or nasal swabs from symptomatic people when taken within the first 5 days, which could allow earlier diagnosis. This was verified in 5 symptomatic people sampled very soon after symptom onset; the nasal and nasopharyngeal samples tested positive later than the saliva samples. There have been a few reports indicating that saliva samples allow earlier diagnosis (5, 17, 27). Whether this could be related to the Omicron variant remains to be assessed, but Lai et al. also reported that saliva samples were more sensitive early in infection in a recently published study performed before Omicron emergence (27). Greater sensitivity early in infection, when transmission is most likely, suggests that saliva samples could help limit virus spread.

The *C_T_* values of NPs samples are lower than those for saliva or ANs samples, indicating a greater viral load. Interestingly, we found similar results for symptomatic subjects sampled within 5 days of symptom onset whereas we could have expected higher viral loads in saliva samples, which performed better. Published data on viral loads of different samples vary, probably because such comparisons are delicate and subject to bias (5, 28–30). Nasal and NP swabs are usually discharged into different volumes of transport medium, while saliva is collected into empty containers. Saliva often requires additional treatment or dilution because of its viscosity.

This study has several limitations. First, it was conducted when only the Omicron variant was circulating in our region, so that it was impossible to compare, within the same time frame, the performance of the tests on Omicron and non-Omicron variants. This is why we compared our present results with those of a previous study (22). While the sample collection/pretreatment methods and the populations studied were similar, other factors could influence our results such as the diagnostic kit used, the screening policy at the time of the study, and the vaccination status of patients. Second, we obtained longitudinal data for only a few subjects. Third, symptoms and vaccination data were self-reported. Lastly, we did not evaluate unsupervised saliva collection.

To conclude, our results provide clinical evidence that saliva samples perform well for detecting the Omicron SARS-CoV-2 variant. In addition to being greatly appreciated by patients, economical, and safer for health care personnel, saliva samples could help limit virus spread as they enable earlier diagnosis.

## MATERIALS AND METHODS

### Subjects.

The subjects were individuals at risk of COVID-19 (symptomatic individuals or asymptomatic contact cases) or previously diagnosed who came to the Toulouse University Hospital drive-through testing center between 2 February and 19 February 2022 during the Omicron surge. Their symptoms, time since symptom onset, and vaccination status were all recorded. They each then provided 3 samples, one each of saliva, anterior nasal swab (ANs), and nasopharyngeal swab (NPs), for COVID-19 tests. Individuals who had discordant SARS-CoV-2 results according to sample type and no previous diagnosis were contacted to perform follow-up analyses for further confirmation. This study was part of the national SARS-CoV-2 surveillance. French law (CSP Art.L1121-1.1) does not require institutional review board approval.

### Specimen collection and processing.

Saliva samples were collected first, supervised by a health care worker. Individuals were instructed not to eat, drink, or smoke 30 min prior to saliva collection. Subjects swilled their saliva around their mouths for at least 30 s before spitting 0.5 to 1 mL of saliva into a sterile container (28). ANs and NPs samples were then collected by trained clinical staff using similar swabs and placed in 3.5 mL virus transport medium (virus sampling kit; Yocon, Beijing, China). ANs samples were collected by inserting a flocked swab about 1 to 1.5 cm into the subject’s nostril. NPs samples were taken from the other nostril following CDC specimen collection guidelines (1). All samples were tested in the virology laboratory within 24 h. Saliva samples were diluted 3-fold in minimum essential medium (MEM) for testing.

### Laboratory testing.

The saliva, ANs, and NPs samples were investigated in parallel. RNA was extracted on an MGI SP-960 instrument using the MGIEasy nucleic acid extraction kit (MGI). An MS2 phage was added to all samples before extraction to validate nucleic acid extraction and PCR, as recommended by the manufacturer. The extracted RNA was amplified on a QuantStudio 5 real-time PCR system (Applied Biosystems) with the Thermo Fisher TaqPath COVID-19 reverse transcription-PCR (RT-PCR) kit that targets sequences in the virus ORF1ab, N, and S genes (31). The Omicron BA.1 variant was identified based on TaqPath S gene target failure (SGTF) or the S gene target late detection (SGTL) profile (12, 32). As the Delta variant was no longer circulating in Toulouse at the time of the study, the Omicron BA.2 variant was identified based on the non-SGTF/SGTL TaqPath profile.

### Statistical analysis.

The sensitivities of saliva, NPs, and ANs samples were calculated using the total number of positive subjects diagnosed with at least one test as reference. The 95% confidence intervals (95% CI) were calculated by the Clopper and Pearson method. Sample type sensitivities were compared using Fisher’s exact tests. We compared the N cycle threshold (*C_T_*) values of saliva, NPs, and ANs of individuals who tested positive in all 3 specimens using Dunn’s multiple-comparison test. A statistically significant difference was defined as a *P* value of <0.05. Statistical analyses were performed using GraphPad Prism 7 (GraphPad Software, Inc.).
